# Is detection of adverse events affected by record review methodology? an evaluation of the “Harvard Medical Practice Study” method and the “Global Trigger Tool”

**DOI:** 10.1186/1754-9493-7-10

**Published:** 2013-04-15

**Authors:** Maria Unbeck, Kristina Schildmeijer, Peter Henriksson, Urban Jürgensen, Olav Muren, Lena Nilsson, Karin Pukk Härenstam

**Affiliations:** 1 Karolinska Institutet, Department of Clinical Sciences, Danderyd Hospital, Division of Orthopaedics, Stockholm, Sweden; 2 School of Health and Caring Sciences, Faculty of Health, Social Work and Behavioural Sciences, Linnaeus University, Kalmar, Sweden; 3 Karolinska Institutet, Department of Clinical Sciences, Danderyd Hospital, Division of Cardiovascular Medicine, Stockholm, Sweden; 4 Qulturum, Jönköping County Council, Jönköping, Sweden; 5 Department of Medical and Health Sciences, Linköping University, Department of Anaesthesia, County Council of Östergötland, Linköping, Sweden; 6 Karolinska Institutet, Medical Management Centre, Stockholm, Sweden; 7 Karolinska University Hospital, Division of Paediatrics, Astrid Lindgren’s Children’s Hospital, Stockholm, Sweden

**Keywords:** Adverse event, Safety, Retrospective record review, Orthopaedic care

## Abstract

**Background:**

There has been a theoretical debate as to which retrospective record review method is the most valid, reliable, cost efficient and feasible for detecting adverse events. The aim of the present study was to evaluate the feasibility and capability of two common retrospective record review methods, the “Harvard Medical Practice Study” method and the “Global Trigger Tool” in detecting adverse events in adult orthopaedic inpatients.

**Methods:**

We performed a three-stage structured retrospective record review process in a random sample of 350 orthopaedic admissions during 2009 at a Swedish university hospital. Two teams comprised each of a registered nurse and two physicians were assigned, one to each method. All records were primarily reviewed by registered nurses. Records containing a potential adverse event were forwarded to physicians for review in stage 2. Physicians made an independent review regarding, for example, healthcare causation, preventability and severity. In the third review stage all adverse events that were found with the two methods together were compared and all discrepancies after review stage 2 were analysed. Events that had not been identified by one of the methods in the first two review stages were reviewed by the respective physicians.

**Results:**

Altogether, 160 different adverse events were identified in 105 (30.0%) of the 350 records with both methods combined. The “Harvard Medical Practice Study” method identified 155 of the 160 (96.9%, 95% CI: 92.9-99.0) adverse events in 104 (29.7%) records compared with 137 (85.6%, 95% CI: 79.2-90.7) adverse events in 98 (28.0%) records using the “Global Trigger Tool”. Adverse events “causing harm without permanent disability” accounted for most of the observed difference. The overall positive predictive value for criteria and triggers using the “Harvard Medical Practice Study” method and the “Global Trigger Tool” was 40.3% and 30.4%, respectively.

**Conclusions:**

More adverse events were identified using the “Harvard Medical Practice Study” method than using the “Global Trigger Tool”. Differences in review methodology, perception of less severe adverse events and context knowledge may explain the observed difference between two expert review teams in the detection of adverse events.

## Background

The “Harvard Medical Practice Study” (HMPS) [1] provided researchers with a retrospective record review method for identifying the incidence of adverse events (AEs) using 18 screening criteria. The method has undergone modifications in subsequent studies [2-6]. The ”Global Trigger Tool” (GTT) method, developed after HMPS and popularised by the Institute for Healthcare Improvement [7], is a method widely used for retrospective reviews in patient safety work. The GTT was primarily designed as a quality improvement tool in to be used in clinical practice for estimating and tracking AE rates over time using 54 triggers [8-14]. The HMPS method is mostly used for research purposes and not in local, routine data collection or for evaluating patient safety interventions in contrast with GTT [15]. Both methods are based on a structured approach to identify events empirically associated with healthcare-related harm. An overview of similarities and differences between the two methods is presented in Table 1.

**Table 1:** An overview of the origins of the “Harvard Medical Practice Study” method and the “Global Trigger Tool”

**Origin and perspective**	**Definition/inclusion and method of review**	**Review stage 1**	**Review stage 2**	**Criterion/trigger**	**Sample size and time frame for inclusion**
**“Harvard Medical Practice Study” (HMPS)**[1,16,17] with subsequent modifications [2-6,18,19]	An unintended injury or complication that results in disability at discharge, death or prolonged hospital stay and is caused by healthcare management rather than the patient’s underlying disease	Generally one reviewer per record	Mostly two physician reviewers per record	General for both methods:	Random, big samples to measure the incidence and to generalise the result
Medicolegal and focus on negligence the first studies and thereafter quality improvement and preventability perspective	Includes both omission and commission	Screening for one of 18 criteria by trained nurses (can be other professionals)	Detailed independent review	An indication that patient harm may have occurred	An AE had to have occurred before and during and detected during and/or after index admission
	Adult, inpatients, often exclusion of e.g. psychiatric and rehabilitation patients	Comprehensive reading	Assess the AE by using different scales according to e.g. causation, severity, preventability, timing, causes, and types	Directs the medical reviewer to relevant parts of the records by the notes	
		No assessment, generally only registration of found criteria, description of the potential AE, and a brief summary of the admission	Generally includes only one AE per patient i.e. the most severe	Some criteria/triggers are AEs by definition e.g. healthcare-associated infections	Different inclusion periods before and after index admission
Research method	Two [1,2] - three [6,18] stage retrospective record review	No time limit	No time limit	Positive criteria/triggers may be without connection to patient harm i.e. false positive	
				HMPS method:	
				18 broad criteria	
**“Global Trigger Tool”**[7]	Unintended injury resulting from or contributed to by medical care that requires additional monitoring, treatment or hospitalisation, or that results in death	Two reviewers per record	The team discuss the findings together	54 triggers, mostly narrow	Random, small samples sufficient for the design of safety work over time
Quality improvement tool for clinical practice	Includes commission, excludes omission	First screening independently for one of 54 triggers by trained nurses (can be other professionals), focus on triggers, no comprehensive reading, reads just relevant parts related to found triggers; second, consensus	One physician, who does not generally review the record but does authenticate the consensus findings of the AEs, the severity rating, and answer questions from reviewers in review stage 1	The Swedish version [20] contains 53 triggers, the triggers “restraint use” in the care module and “other” in the medication module were excluded, and a trigger, “occurrence of any postoperative complication“, was added in the surgical module	10 records every second week or 20 records every month per hospital
Track AE rate over time in a hospital or a clinic	Adult, inpatients, exclusion of psychiatric and rehabilitation patients	Finds triggers, describes the potential AE, and categorise harm according to NCC MERP index [21]	The physician is the final arbitrator		Length of stay at least 24 hours
	Two stage retrospective record review	No assessment of preventability	All identified AEs are included		An AE had to have occurred before and during and detected during and/or after index admission
		Maximum 20 minutes per record	No time limit		30 days inclusion period before and after index admission
			The Swedish version includes the same preventability scale as used in HMPS methodology [2,20]		

AE, adverse event; NCC MERP, National Coordination Council for Medication Error Reporting and Prevention (NCC MERP) index [21]. Category A-D describes risk and no harm incident. Category E-I describes harm.

There has been a theoretical debate as to which retrospective record review method is the most valid, reliable, cost efficient and feasible [15]. A limitation when comparing different retrospective record review studies is that the AE definition, inclusion frame, settings and the presentation of the AE rates have varied. It is of interest to evaluate the feasibility and capability of using different retrospective record review methods to identify AEs at departmental level since record review is increasingly used in local patient safety initiatives. To the best of our knowledge, the HMPS method and the GTT have not previously been evaluated on the same sample.

The aim of the study was to evaluate the feasibility and capability of the HMPS method and the GTT to identify AEs in a specified clinical setting.

## Methods

### Setting and sampling

The study was performed at a university hospital in the Stockholm metropolitan area, with a four-ward, 52-bed, orthopaedic department, including both elective and acutely admitted inpatients.

All orthopaedic inpatient admissions during 2009 (*n*=3701) were available for randomisation, irrespective of length of stay. Based on earlier studies [2,3,22-24] we estimated that AEs would occur in 16% of admissions and calculated that a sample of 350 admissions would be sufficient to estimate the prevalence of AEs with a 95% CI of ± 3.8%.

### Definitions and inclusion criteria

For both methods an AE was defined as an unintended harm to the patient that was caused by healthcare rather than by the patient’s underlying disease process. In contrast to earlier HMPS method studies [1,5,6,18,25], we did not require that the AE prolonged the hospital stay or caused disability at the time of discharge. Both acts of omission and acts of commission were included. A preventable AE was defined as an error in healthcare management due to failure in following accepted practice at the level of the individual or of the system [2,4].

The orthopaedic admission in the random sample constituted the index admission. To be included in the study, the AE had to be related to care given in the Orthopaedic Department and, additionally, one of the following criteria had to be met;

(i) The AE had to have been caused within 30 days before index admission, leading to the index admission, or to have been detected during the index admission.

(ii) The AE had to have been caused and detected during index admission.

(iii) The AE had to have been caused during index admission and have been detected within 30 days of index discharge from the Orthopaedic Department. AEs identified using this criterion were not required to result in a new admission; e.g., a postoperative deep vein thrombosis treated on an outpatient basis was included.

Index admissions due to deep arthroplasty infections were included as AEs up to one year after surgery.

The computerised medical record system in which all documentation was made by healthcare personnel and departments at the hospital is referred to as the record. Both inpatient and outpatient notes were included. Screening criteria are referred to as criteria.

### Description of the teams

Two teams, one for each method, made the reviews. Each team consisted of one registered nurse (RN) and two physicians; all were used to working with patient safety issues. The HMPS method team consisted of a senior RN with wide experience of using criteria and knowledge about the orthopaedic context including the computerised record system. One physician was a senior orthopaedic surgeon with experience in record review. The other physician was junior without orthopaedic context knowledge and review experience but expertise in the field of patient safety.

The whole GTT team had extensive experience in using the GTT method. The team consisted of senior reviewers and even though they had been trained in other specialties they were experienced in reviewing records from different medical specialities including orthopaedics and using other computerised record systems.

### Standardisation of the review process before study start

To standardise the review process a written manual, including definitions and detailed examples for each method was developed, discussed and approved by all reviewers before the start of the study. During the process of familiarisation, each team member independently reviewed 11 training records on paper and a consensus process allowed discussions of the AE assessments and related matters. Team members not familiar with the specific computerised record system received one hour’s training in theory. In order to further familiarise with the new record system the nurse reviewer of the GTT team read seven computerised training records before starting the review process. Questions about the record system and local routines could be asked when needed during the review process.

### Review process including evaluation

A two-stage retrospective record review was performed.

**Figure 1:**
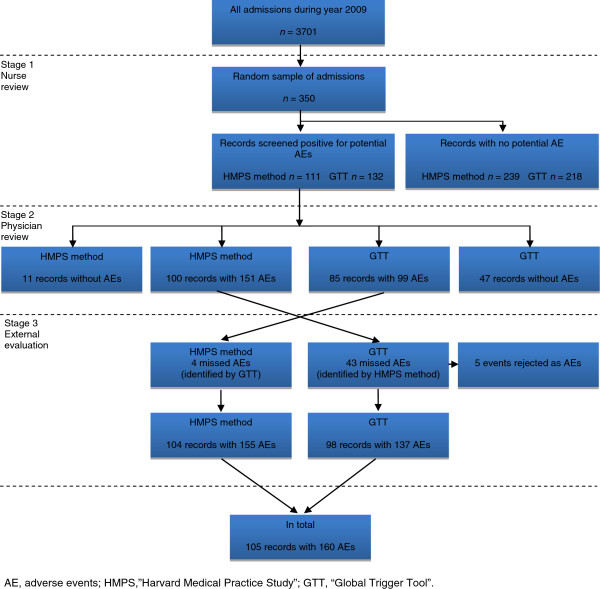
Three-stage review process for detecting adverse events.

In review stage 1, all records in the random sample were reviewed by the RNs, one for each method (Figure 1). They screened for the presence of one or more of 18 predefined criteria or 53 triggers, respectively. For every criterion/trigger detected, a judgement was made by the RN regarding whether the criterion/trigger reflected the presence of a potential AE or not and the potential AE was described in brief. The time taken to review each record was documented. Only records with potential AEs were forwarded to the physicians for review. Each physician in the respective teams reviewed half of the records forwarded by the RN.

In review stage 2, the physicians performed an independent review (Figure 1). A judgement was made regarding whether patient harm had occurred or not. An assessment of healthcare causation was performed using a 6-point scale [1,2]. A score of four or higher (i.e. more than 50% likelihood of healthcare causation) was regarded as being an AE. A similar 6-point scale was used to judge the preventability of the AE. A score of four or more meant that the AE was considered as having been preventable [2]. The severity of the AE was judged using standardised scales for the method being used, since each method has its own severity scale [1,21]. In addition, all physicians documented which criterion/trigger was related to each AE. If a record contained more than one AE, each was reviewed separately and the associated criterion/trigger was documented separately. The time taken to do the review of each event was documented. The HMPS method team made additional assessments about the nature of the AEs according to the methodology. In addition to reviewing every second forwarded record, a random sample of the forwarded records was reviewed double-blinded to assess inter-rater reliability between the physicians´ judgements within each team. After independent review of all potential AEs, the physicians in each team discussed the duplicate reviewed records and reached consensus.

We evaluated both teams´ nurse review processes. First, in review stage 2 the physicians included any additional AE they found that had not been identified by the nurse reviewer in stage 1. Furthermore, every tenth record that had been deemed as not containing a potential AE in the nurse review process was screened for AEs by one of the physicians in each team.

In a third review stage all AEs that were found with the two methods together were compared and all discrepancies after review stage 2 were analysed by one of the RNs (MU). Events that had only been identified by one of the methods were reviewed by the physicians within the other team. The aim of the third review stage was twofold: the first was to employ the external evaluation of both the methods to reach the “true documented and confirmed AE rate in the sample”, and the second aim was to compare the physicians’ judgements in the full set of identified AEs (Figure 1).

### Statistical analysis

Categorical data are summarised using frequency counts and proportions (percent). Both the number of AEs per record and the number of records with at least one AE are presented. The calculation of 95% confidence interval for a proportion is based on exact methods according to Clopper-Pearson. Continuous data are presented as median and range/inter-quartile range. The inter-rater reliability was calculated using binary data. The Spearman rank order correlation coefficient (*r*_s_) was used to investigate “learning curves”. P<0.05 was considered statistically significant. Statistical programs used to collate the results were Excel and Statistica 9.0.

### Ethics approval

Ethics approval was provided by the regional Ethics Committee of Stockholm (number 2008/951-31/3).

## Results

### The review process

No records were excluded from review due to missing documentation.

The study sample, representing 1848 hospital days, comprised a majority of female patients (*n*=201, 57%) and acutely admitted patients (*n*=250, 71%). The median (interquartile range) age was 69 (53–81) years and length of stay was four (2–7) days.

In total, 160 different AEs were identified in 105 (30.0%) of the 350 records with both methods combined after the third review stage (Figure 1).

In review stage 1, 111 (HMPS method) and 132 (GTT) of the 350 records contained a potential AE and were forwarded to physician review (Figure 1).

After review stage 2, the HMPS method found 151 (94.4%, exact 95% CI: 89.6-97.4) AEs in 100 (28.6%) of 350 records, resulting in a mean rate of 0.43 AEs per record (range 0–5), of which 131 (86.7%) were deemed preventable. Using the GTT, the physicians identified 99 AEs (61.9%, exact 95% CI: 53.9-69.4) in 85 (24.3%) of the records, on average 0.28 AEs per record (range 0–4), and 77 (77.8%) of these AEs were deemed preventable (Figure 1).

The nurse reviewer validation steps did not identify any additional AEs for the HMPS method and identified nine AEs for the GTT.

Four AEs not identified by the HMPS method and 43 not identified by the GTT were forwarded to the third review stage. After the third review stage, 155 (96.9%, exact 95% CI: 92.9-99.0) and 137 (85.6%, exact 95% CI: 79.2-90.7) AEs were identified in 104 (29.7%) and 98 (28.0%) of the 350 records by using the HMPS and GTT methods, respectively. Of these AEs, 135 (87.1%) (HMPS) and 110 (80.3%) (GTT) were deemed preventable by the physicians.

Thirty (HMPS), and 43 (GTT) potential AEs were reviewed in duplicate by the physicians. The physicians’ initial assessments before team discussions considering healthcare causation were coherent within the teams in 93% and 88% of the cases for the HMPS and GTT methods, respectively, and preventability in 100% and 95% of the cases, respectively.

The median (range) time for each record for nurse review was three (1–35) versus eight (1–20) minutes for the HMPS and GTT methods, respectively. A negative correlation between nurse review time and serial samples number was demonstrated for both methods, r_s_ for the HMPS method was −0.36, (*p*<0.001), and r_s_ for GTT was −0.44, (*p*<0.001), indicating a “learning curve” for both RNs. The median review time for both physician reviewers using the HMPS method was six minutes (range 2–18 and 2–31 minutes). In the GTT team, median times were four and eight minutes (range 1–15 and 2–30 minutes).

We analysed the AEs not identified after review stage 2 (*n*=4 (HMPS) and 43 (GTT)). For GTT there were no differences in types and rates of unidentified AEs irrespective of whether assessment was performed early or late in the review process. The description, nature and potential underlying causes to the missed AEs for both methods after review stage 2 are presented in Table 2.

**Table 2:** Description, nature and known potential underlying causes for the missed AEs after review stage 2

**Missed AEs by the HMPS method**	**Rejected as AE**	**Severity scale**^**a**^	**Criteria number**^**b**^	**Orthopaedic knowledge**	**Computerised record system knowledge**	**Time limit 20 minutes**
Pressure ulcer		1	3			NA
Pain		1	1			NA
Infiltrated intravenous infusion		1	3			NA
Pressure ulcer		1	3			NA
**Missed AEs by the GTT**	**Rejected as AE**	**Severity Scale**^**c**^	**Triggers C13 procedure” and/or C14 care: other”**	**Orthopaedic knowledge**	**Computerised record system knowledge**	**Time limit 20 minutes**
Re-fracture		G		X		X
Urinary retention		E	X			
Neurological harm		E	X	X	X	
Wound infection		F			X	
Wound infection		E			X	
Pulmonary embolism		F				
Infiltrated intravenous infusion		E	X			
Infiltrated intravenous infusion		E	X			
Urinary retention		E	X			
Wound infection		F			X	
Urinary retention		E	X			
Discharge deficiencies		F				
Urinary tract infection		F	X			
Urinary retention		E	X			
Urinary retention		E	X			
Thrombophlebitis		E	X			
Pressure ulcer		E				
Late detection of fracture		F	X			
Mistreated fracture		F	X	X		
Wound during dressing		E	X			X
Urinary retention	X					
External fixation, wound		E	X			X
Red skin from bandage		E	X			
Fungus		E				
Pressure ulcer		E				
Urinary retention		E	X			
Infiltrated intravenous infusion		E	X			
Ad latus displaced fracture	X					
Sensory loss		E	X	X		
Overdose of drug		F	X			
Heparin induced thrombocytopenia		E	X			X
Rash due to bandage		E	X			
Pressure ulcer		E				
Pressure ulcer		E				
Wound infection		E				
Rash due to drug		E	X			
Bleeding during surgery	X					
Increased liver enzymes	X					
Subsidence of the femoral stem	X					
Wrong operation performed initially		F				
Urinary retention		E	X			
Neurological harm		G	X	X		
Bleeding when urinary catheter is removed		E	X			X

AE, adverse events; HMPS, “Harvard Medical Practice Study”; GTT,” Global Trigger Tool”; NA, Not Applicable for this method.

^a^ 1, minimal impairment, recovery within 1 month.

^b^ 1, the index admission was an unplanned admission related to previous healthcare management within 30 days; 3, hospital-incurred patient injury.

^c^ E, contributed to or resulted in temporary harm to the patient and required intervention; F, contributed to or resulted in temporary harm to the patient and required initial or prolonged hospitalisation; G, Contributed to or caused permanent patient harm.

### Positive predictive value

A total of 466 (range: 1–11 per record) criteria and 737 (range: 1–13 per record) triggers were identified in 195 (HMPS) and 233 (GTT) records after the third review stage. Criteria and triggers varied in frequency from common to never detected (Tables 3 and 4). Positive predictive value (PPV) of criteria and triggers was defined as the number of times a specific criterion/trigger identified an AE divided by the total number of times the criterion/trigger was found. Individual criteria and triggers varied in their yield of detections of AEs and the percentage (range) of PPV was 40.3% (0.0-80.0) for the HMPS method and 30.4% (0.0-100.0) for the GTT (Tables 3 and 4).

**Table 3:** Outcome of the respective criterion in relation to the adverse event

**Criteria**	***n *(%) of positive criteria**	***n *of criteria related to AE**	**PPV AE**
Hospital-incurred patient injury	132 (28.3)	83	62.9
Any other undesirable outcome not covered above	87 (18.7)	8	9.2
Unplanned re-admission after discharge from index admission within 30 days*	85 (18.2)	25	29.4
Healthcare-associated infection or sepsis	38 (8.2)	22	57.9
Adverse drug reaction	33 (7.1)	12	36.4
The index admission was an unplanned admission related to previous healthcare management within 30 days	26 (5.6)	13	50.0
Dissatisfaction with care documented in the patient’s medical record	16 (3.4)	2	12.5
Other patient complication	13 (2.8)	7	53.8
Unplanned return to the operating room	10 (2.1)	2	20.0
Unplanned removal, injury or repair of an organ during surgery	5 (1.1)	4	80.0
Unplanned transfer from general care to intensive care	5 (1.1)	4	80.0
Development of neurological deficit not present on admission	5 (1.1)	3	60.0
Inappropriate discharge to home	5 (1.1)	1	20.0
Documentation or correspondence indicating litigation	3 (0.6)	1	33.3
Cardiac or respiratory arrest	2 (0.4)	1	50.0
Unplanned transfer to another acute care hospital	1 (0.2)	0	0.0
Unexpected death	0 (0.0)	0	0.0
Injury related to abortion or delivery	NA		
Total	466 (100)	188	40.3

AE, adverse events; PPV, positive predictive value; NA, not applicable.

All criteria except 1, 2 and 4 must occur during index orthopaedic admission to be included as positive criteria.

* Including outpatient visits.

**Table 4:** Outcome of the respective trigger in relation to the adverse event

**Triggers**	***n *(%) of positive triggers**	***n *of triggers related to AE**	**PPV AE**
Procedure	117 (15.9)	25	21.4
Care: other	106 (14.4)	30	28.3
Anti-emetic administration	74 (10.0)	1	1.4
Abrupt drop in haemoglobin	66 (9.0)	2	3.0
Occurrence of any postoperative complication	64 (8.7)	49	76.6
Healthcare-associated infections	60 (8.1)	34	56.7
Re-admission within 30 days	59 (8.0)	13	22.0
Pressure ulcers	33 (4.5)	25	75.8
Return to surgery	21 (2.8)	8	38.1
Falls	16 (2.2)	3	18.8
Transfusion of blood or use of blood products	15 (2.0)	1	6.7
Abrupt medication stop	14 (1.9)	2	14.3
Change of anaesthetic during surgery	13 (1.8)	1	7.7
Removal/injury or repair of organ during operative procedure	10 (1.4)	3	30.0
Vitamin K administration	9 (1.2)	0	0.0
Change in procedure	8 (1.1)	5	62.5
X-ray or Doppler studies for emboli or deep vein thrombosis	7 (0.9)	6	85.7
Codes or arrest	7 (0.9)	3	42.9
Transfer to higher level of care	6 (0.8)	4	66.7
Re-admission to the Emergency Department (ED) within 48 hours	6 (0.8)	1	16.7
Diphenhydramine administration	4 (0.5)	1	25.0
Over-sedation/hypotension	3 (0.4)	2	66.7
Insertion of arterial or central venous line during surgery	3 (0.4)	0	0.0
Post-operative increase in troponin levels	2 (0.3)	2	100.0
Intubation/reintubation / BiPaP in post anaesthesia care unit	2 (0.3)	0	0.0
Consult requested in post anaesthesia care unit	2 (0.3)	0	0.0
Time in ED greater than 6 hours	2 (0.3)	0	0.0
Naloxone administration	2 (0.3)	1	50.0
Clostridium difficile positive stool	1 (0.1)	1	100.0
Admission to intensive care post-operatively	1 (0.1)	1	100.0
Positive blood culture	1 (0.1)	0	0.0
International Normalised Ratio (INR) greater than 6	1 (0.1)	0	0.0
Glucose less than 3 mmol/litre	1 (0.1)	0	0.0
Rising BUN or serum creatinine two times (2x) over baseline	1 (0.1)	0	0.0
Total	737 (100)	224	30.4

*AE*, adverse events; *PPV*, positive predictive value.

None of the triggers in the Intensive Care Module (pneumonia onset, readmission to intensive care unit, in-unit procedure or intubation/re-intubation) were identified in this study.

None of the following triggers were identified in this study; In hospital stroke, dialysis, X-ray intra-operative or in post anaesthesia care unit, intra- or postoperative death, mechanical ventilation greater than 24 h post-operatively, intra-operative administration of Epinephrine, Norepinephrine, Naloxone or Flumazenil, pathology reports normal or identifying specimen unrelated to initial surgical diagnosis, operative time greater than 6 h, partial thromboplastin time greater than 100 seconds, and Flumazenil administration.

The five GTT perinatal triggers were not applicable in this study.

### Harm

Most AEs, irrespective of the method used, resulted in minor, transient harm and the majority were judged to be preventable (Table 5). The main difference between the methods regarding severity and types of AEs was found among the ones causing minimal or moderate impairment (Table 2). These were predominantly urinary retention, infiltrated intravenous infusions, pressure ulcers and healthcare-associated infections.

**Table 5:** Severity and preventability of adverse events

**Category of harm HMPS method**	**HMPS method after review stage 2**		**HMPS method after review stage 3**	
	***n *(%) of AEs**	***n *(%) of preventable AEs**	***n *(%) of AEs**	***n *(%) of preventable AEs**
Minimal impairment, recovery within 1 month	116 (76.8)	104 (89.7)	120 (77.4)	108 (90.0)
Moderate impairment, recovery within 1 to 6 months	20 (13.2)	17 (85.0)	20 (12.9)	17 (85.0)
Moderate impairment, recovery within 6 to 12 months	9 (6.0)	6 (66.7)	9 (5.8)	6 (66.7)
Permanent impairment, degree of disability <50%	4 (2.6)	3 (75.0)	4 (2.6)	3 (75.0)
Permanent impairment, degree of disability >50%	1 (0.7)	1 (100.0)	1 (0.6)	1 (100.0)
Contributed to patient death	1 (0.7)	0 (0.0)	1 (0.6)	0 (0.0)
Unable to determine	0 (0.0)	0 (0.0)	0 (0.0)	0 (0.0)
Total n of AE	151 (100.0)	131 (86.8)	155 (100.0)	135 (87.1)
**Category of harm GTT**	**GTT after review stage 2**		**GTT after review stage 3**	
	***n *(%) of AEs**	***n *(%) of preventable AEs**	***n *(%) of AEs**	***n *(%) of preventable AEs**
E Contributed to or resulted in temporary harm to the patient and required intervention	51 (51.5)	41 (80.4)	78 (56.9)	63 (80.8)
F Contributed to or resulted in temporary harm to the patient and required initial or prolonged hospitalisation	42 (42.4)	31 (73.8)	51 (37.2)	40 (78.4)
G Contributed to or caused permanent patient harm	5 (5.1)	5 (100.0)	7 (5.1)	7 (100.0)
H Intervention required to sustain life	1 (1.0)	0 (0.0)	1 (0.7)	0 (0.0)
I Contributed to patient death	0 (0.0)	0 (0.0)	0 (0.0)	0 (0.0)
Total n of AE	99 (100.0)	77 (77.8)	137 (100.0)	110 (80.3)

AE, adverse events; HMPS, “Harvard Medical Practice Study”; GTT, “Global Trigger Tool”.

## Discussion

The aim of the present study was to evaluate the feasibility and capability of the HMPS method and the GTT in being able to identify AEs in a specified clinical setting. To the best of our knowledge, this is the first study that explicitly evaluates these common retrospective record review methods in the same sample and with the same definitions. In total 160 AEs were found in 105 (30.0%) of 350 records. The HMPS method confirmed 13% more AEs after the third review stage. The main difference was found among AEs causing minor or moderate impairment.

A first interpretation might be that the differences in the AE rate after review stage 2 depended on the restriction of review time to 20 minutes in the GTT method. However, only five AEs may have been unnoticed as a consequence of this restriction. Furthermore, the nurse review time using the HMPS method was less than for the GTT. Searching for triggers in different parts of the record may take longer time than just comprehensively reading the text and searching for broad criteria in orthopaedic care comprising short hospital stays and limited documentation. Record review has been criticised as being too time consuming and therefore too expensive [26]. Our results show that the review times for experienced RNs and physicians are short and were shorter than, or similar to, those in other studies [15,27-30]. The length of hospital stays, for example, most probably affects the review times, making comparisons between studies difficult. If retrospective record review can identify more AEs than traditional incident reporting methods, the time used for review may be cost-effective [8].

Another explanatory factor for differences in the AE rates may be the perception of AEs. The distinction between a no-harm incident and a less severe AE is not sharp and is subject to the individual assessments, which may affect the outcome of a review process, irrespective of method used, study design or consensus. Even if studies are well-planned, definitions and scales may not be fully clear. A manual cannot describe all conceivable AEs because situational and individual factors must be applied in the implicit review. Experience of the specific record system and local context, may have affected the numbers of identified potential AEs in review stage 1. The discrepancy in AEs between review stages 2 and 3 could be due to differences in the two review methods. This study also included less severe AEs e.g. infiltrated intravenous infusions. According to the GTT method, at least as interpreted by our expert reviewers before the start of the study, these were not considered as being AEs as they often did not require any intervention. The difference in AE rate after review stage 3 showed that the physicians in the GTT team were more likely to reject minor events as AEs than those in the HMPS method team. Classen et al. [8] have found that the greatest variability between the reviewers in severity categorising of AEs was related to the lowest harm level in the severity scale used in GTT, category E. The perception of minor AEs affects the review outcome but also subsequently the organisation’s input regarding learning about good safety practice. Olsen [15] stated that even if an AE caused only minor physical harm, it may still be detrimental to the patient’s psychological recovery, participation and trust. Another reason to treat minor AEs as important is because an AE that causes only minor harm in one instance might be a sentinel of serious system defects that could result in major harm in the next case. Patient safety interventions are needed to reduce major as well as minor AEs.

We chose to have a common AE definition since the scope of the study was to evaluate the two methods. The AE definition used in the present study was broad and did not require, in contrast to the original HMPS method, that the patient should have experienced any disability or prolonged hospital stay as a result of the AE. Several HMPS method studies have only included the most severe AE per record. The original GTT method includes all AEs that cause physical harm and this approach was also used in our study (Table 1). Consequently, the AE definition used by the original GTT method may be more sensitive to minor harms than those of the HMPS method. However, the severity scale used in the GTT [7] required an intervention to be present to qualify as a minor AE (category E). The perception of an intervention could have affected both the AE rate and the inter-rater reliability outcome within the GTT team. Apart from the AE definition, the severity scale used in the HMPS method is, by contrast, more inclusive of minor AEs [2,6]. One of the lessons learned from the study is that the methods can be used with different AE definitions than those used originally in the descriptions of the respective method [1,7].

Individual criteria and triggers varied in their yield of identified AEs. In this study, some were always associated with AEs, while others were never associated. Some of the criteria and triggers were irrelevant for orthopaedic care and some were not identified in our sample. In a study [10] with a larger sample, some triggers were seldom or never observed, which is in agreement with our experience. However, the non-specific criterion “any other undesirable outcome not covered above” and the triggers “procedure” and “care: other” were commonly used and may indicate the necessity for providing more descriptions with examples in the manual in order to create a more valid and reliable review process. The PPV is also affected by the larger number of triggers compared to the number of criteria. Criteria are indefinite about the type of AE that affected the patients outside index admissions. This has affected the total PPV positively.

The preventability rates were considerably higher in this study than those reported in other surgical studies [31-33] and are more comparable to the preventability outcome in the Swedish national AE study [6]. This may be due to the use of experienced reviewers and to the condition that all reviewers were used to working with patient safety issues leading to a patient safety perspective in the review process. Another reason could be that many nursing related AEs were identified in the present study, for example pressure ulcers and urinary retention, and those are often judged as being preventable.

Irrespective of method applied, it is important in clinical patient safety work to have stable internal review teams with context knowledge that can lead them to develop greater expertise and who also produce consistent reviews, if trends are to be detected in less review time [11]. Sharek et al. [11] found that an experienced review team identified substantially more AEs than newly trained internal and external teams did. To use retrospective record review at a department level and to develop the review process by, for example, categorising the nature of the AEs according to the HMPS methodology may be important steps in increasing local safety learning and involvement. The median review time for the physicians using the HMPS method was the same as the physicians in the GTT team leading to the conclusion that categorising the nature of AEs does not take additional review time. This knowledge can be used to guide local limited improvement resources into specific areas and/or processes where tailored interventions and redesign are necessary to create resilience [13,34].

### Limitations

Our study has several limitations besides those traditionally associated with retrospective record review, for example, the AE occurrence, severity and preventability can be overestimated due to hindsight bias; but it can also be underestimated, due to, for example, incomplete documentation. Context knowledge including knowledge about the computerised record system may have affected parts of the outcome. We had one team per method. A cross over methodology could have reduced possible bias in this aspect. Furthermore, a study including more teams, teams with other skills or records from other medical specialities may have given different results. This study was limited to hospital orthopaedic AEs as the aim was to evaluate the methods at a local level. This affects the ability to generalise our findings to other areas of healthcare like elderly medical care with voluminous notes and most probably longer review times.

### Conclusions and future implications

Retrospective record review is a valuable tool in the identification of patient safety deficiencies. More AEs were identified by using the HMPS method than the GTT when compared to the total number of identified AEs. Hospital or orthopaedic context knowledge, methodology including restriction of review time and perception of minor AEs may have affected the results among expert reviewers. If this perception of minor AEs is related to the individuals within the teams or to the methodology itself is unclear. More studies are needed to further assess different sources of safety information in healthcare as well as the evaluation of the capability of different methods to provide decision-makers and staff with an accurate knowledge of system weaknesses and underlying patient safety problems.

## Abbreviations

AE: Adverse event; HMPS: “Harvard Medical Practice Study”; GTT: “Global Trigger Tool”; RN: Registered nurse; PPV: Positive predictive value; NA: Not applicable.

## Competing interests

None of the authors have competing interests. Financial support was provided through the Swedish Vinnvård programme for MU and KPH. Vinnvård has neither been involved in any part of the study, nor in writing the manuscript or in the decision to submit the manuscript for publication.

## Authors’ contributions

MU: Main author, project supervisor, responsible for study design, acquisition of data, analysis, manuscript drafting and intellectual content. KS: Responsible for study design, acquisition of data, manuscript drafting and intellectual content. PH: Responsible for study design, manuscript drafting and intellectual content. UJ: Responsible for study design, acquisition of data and intellectual content. OM: Responsible for study design, acquisition of data, manuscript drafting and intellectual content. LN: Responsible for study design, acquisition of data, manuscript drafting and intellectual content. KPH: Responsible for the study design, acquisition of data, manuscript drafting and intellectual content. All authors read and approved the final manuscript.
